# HPV and Genital Warts among Peruvian Men Who Have Sex with Men and Transgender People: Knowledge, Attitudes and Treatment Experiences

**DOI:** 10.1371/journal.pone.0058684

**Published:** 2013-03-14

**Authors:** César R. Nureña, Brandon Brown, Jerome T. Galea, Hugo Sánchez, Magaly M. Blas

**Affiliations:** 1 Escuela de Antropología, Universidad Nacional Mayor de San Marcos, Lima, Peru; 2 Department of Population Health and Disease Prevention, University of California Irvine, Irvine, California, United States of America; 3 Epicentro, Lima, Peru; 4 Epidemiology, STD and HIV Unit, School of Public Health and Administration, Universidad Peruana Cayetano Heredia, Lima, Peru; University of Washington, United States of America

## Abstract

**Background:**

Several studies have assessed the epidemiology of HPV infection among MSM, but no qualitative studies have specifically assessed how HPV and genital warts (GW) affect South American men who have sex with men (MSM) and male-to-female transgendered women (TG). This study explored the knowledge, attitudes and experiences of Peruvian MSM and TG regarding HPV and GW.

**Methods:**

We performed a qualitative study consisting of fifteen in-depth interviews and three focus groups carried out in Lima, Peru with diverse MSM and TG groups, including sex workers. Resulting data were analyzed by applying a systematic comparative and descriptive content analysis.

**Results:**

While knowledge of HPV was limited, awareness of GW was common, particularly among TG persons and sex workers. Still, few participants recognized that GW are sexually transmitted, and many had problems differentiating between GW and other STI/anogenital conditions. Stigmatizing experiences were common during sexual encounters with people who had visible GW. Shame, emotional and physical troubles, and embarrassing sexual experiences were reported by individuals with GW. Search for treatment was mediated by peers, but stigma and apparent health services’ inability to deal with GW limited the access to effective medical care.

**Conclusions:**

In Peru, public health interventions should strengthen services for HPV/GW management and increase accurate knowledge of the transmission, treatment, and sequelae of HPV/GW in MSM and TG populations.

## Introduction

Human papillomavirus (HPV) is one of the most common sexually transmitted infections (STI) worldwide and it causes morbidity and mortality in both men and women via cervical cancer, penile and anal cancer, oropharyngeal cancer and genital warts (GW) [1]. In many countries HPV prevalence is similar among men and women, with differences dependent on risk factors and methods used to detect infection [1]–[2]. While much of the emphasis in the literature has focused on women and the link between HPV and cervical cancer, evidence is mounting regarding the high prevalence of HPV infection in males, particularly in anogenital sites, and especially in men who have sex with men (MSM) [3]. Studies from developed countries have found a high prevalence of HPV and anal lesions among MSM [3] but data from developing countries are also emerging. For example, a recent study in Peru among 105 MSM found that 77.1% were infected with HPV of which nearly half – 47.3% -- were infected by a carcinogenic type [4]. Similarly, a study in Argentina which included a sample (N = 114) of transgendered (TG) sex workers reported an anal HPV prevalence of 97% and high-risk genotypes were detected in 87.5% of participant samples from which the infecting genotype was determined [5]. While it is well established that anogenital warts are caused by HPV and that HPV is linked to oral, anal and penile neoplasms [6], HPV infection has also been associated with acquisition of HIV in MSM [7]–[9], and there are ongoing studies looking at the association between GW and HIV [10].

Unfortunately, there is little public awareness about the HPV infection, and perhaps less-so in high risk groups [11]. Existing research has focused on women’s limited knowledge and susceptibility to HPV infection and its sequelae, most notably, cervical cancer [11]; however, there has been less empirical work examining men’s knowledge, attitudes and experiences regarding HPV infection and its disease outcomes [12]–[15]. Additionally, highly vulnerable populations such as TG and MSM who perform sex work remain under-represented in the studies on this topic.

In addition to the physiological consequences of HPV-related GW, research is emerging with regards to the social and psychological implications of HPV including its negative impact on quality of life, mental wellbeing and sexual practices [16]–[17], [18]. One study [19] explored the experience of having GW among MSM, concluding that MSM need to be appropriately informed about all aspects of GW, with the aim of alleviating the psychological distress associated with the disease and to optimize preventive efforts and safe sexual behaviour.

Of course, these aspects need to be considered in the light of a major context in which social exclusion, limitations of the health system and STI-related stigma affect the access of MSM/TG populations to health care. For example, recent studies in Peru have found that, in these groups, fear of a positive HIV result and lack of awareness of places where to get tested are important reasons for not taking an HIV test [20]; and that conditions of social vulnerability define for them a situation of high risk and prevalence of HIV and other STI, especially among TG people [21]–[22].

In this paper we present findings of a qualitative study aimed to explore the knowledge, attitudes and experiences of Peruvian MSM and male-to-female TG regarding HPV and GW.

## Materials and Methods

### Participants

Recruitment was based on convenience sampling conducted in Lima, Peru by peer outreach workers in a gay men’s community health center, using snow-ball sampling and venue-based recruitment in places where MSM and TG socialize. Outreach activities were targeted to individuals with diverse sexual identities and behaviors in order to have a heterogeneous sample and different points of view: self-identified “gay” men, male-to-female TG women, men not identifying as “gay” who reported having sex with men and TG sex workers were explicitly sought due to high presence of commercial sex activities in these populations, especially among Peruvian TG [23]. Potential participants were informed of the study objectives, risk and benefits of participation. Interested individuals were referred to the study site for eligibility screening criteria (at least 18 years of age and reporting sex with another male in the previous 12 months). Participants were provided with a verbal consent form signed by the Investigator in their presence once all questions were addressed. Eligible and willing participants were randomly assigned to either a focus group discussion or an in-depth interview. Participants were compensated with 15 Nuevos Soles (approximately US$ 5.6 in 2011) for transportation following study participation. The Institutional Review Board at *Universidad Peruana Cayetano Heredia* approved the study protocol and verbal consent process prior to implementation. Verbal consent was obtained in place of written consent for the protection of the participants in the focus groups and interviews. No names and signatures were recorded elsewhere, as this would have provided identifiable data of participants. Once the Investigator and participant reviewed the verbal consent, and all participant questions and doubts were addressed, the investigator signed the consent form in the presence of the participant. A copy of the verbal consent was provided to the participant. The verbal consent procedure was approved by the ethics committee on February 9, 2011 prior to any participant contact.

### Procedures

#### Focus Groups

The focus groups were primarily aimed to obtain socially shared ideas regarding HPV and GW (commonly held opinions, stereotypes and experiences that participants were able to publicly express) and the group nature may stimulate new ideas or uncover information that may be lost in in-depth interviews [24]. All study procedures were carried out in private places and participants remained anonymous. Three focus groups of 6–8 individuals were convened of persons who self-identified as: 1) gay men; 2) non-“gay” identifying men who reported sex with men; and 3) transgender women (many of whom were sex workers). Focus groups lasted approximately one hour and were conducted in Spanish by two psychologists experienced in HIV/STI prevention with MSM and TG. The facilitators followed a semi-structured focus group guide including themes such as knowledge on HPV and GW, social and community concerns, and attitudes and experiences related to GW. Images of anogential GW were shown to group participants in order to ensure an understanding of GW and to encourage discussion among participants.

#### In-depth interviews

Individual in-depth interviews were carried out to obtain personal visions and accounts on the research topic, for which confidence building was a critical issue during the procedure. One of the discussion group facilitators conducted fifteen interviews. These included participants who self-identified as either gay men (including one sex worker) [N = 6]; non-“gay” identifying men who reported sex with men [N = 4]; and transgender women (including four sex workers) [N = 5]. In-depth interviews were conducted until saturation was achieved, i.e., until no new information was emerging in the interviews and this therefore determined the final number of interviews performed. A semi-structured guide including questions on personal perspectives and experiences regarding GW was used to guide the interviews.

### Data Analysis

Focus groups and interviews were audio recorded and transcribed verbatim. A Peruvian anthropologist experienced in sexuality and STI research (CRN) applied systematic comparative and descriptive content analysis that consisted of grouping and coding the information in thematic categories, and identifying recurring issues and differences in the narratives. A second reviewer (JG) confirmed the analysis and discrepancies were resolved. Representative quotes were extracted and translated into English.

## Results

### Demographics

We recruited 36 participants comprised of three focus groups (of 6–8 participants in each sub-group) and 15 in-depth interviews. The mean participant age was 26 (range 18–40). We did not ask participants if they personally had GW; nevertheless, 4/15 of the in-depth interview participants spontaneously reported having HPV, and the results presented on personal experiences of having GW are based on the information provided by these subjects.

### Focus Groups and In-depth Interviews

Three main themes emerged across the focus group and in-depth interviews: 1) Knowledge of HPV and genital warts; 2) Genital wart-related attitudes and experiences; and 3) Management of genital warts. Each theme is presented below with representative quotes.

### Knowledge of HPV and genital warts

Unfamiliarity with HPV was common though a few participants recognized that HPV affects both men and women or linked GW to HPV. Some participants had heard of the term “papilloma”, a few reported that HPV was a transmissible and incurable infection, and others had little knowledge of HPV and associated it with women’s health problems:

What I’ve heard [about papilloma] had to do with a case that happened to a female Brazilian model whose entire [sex] organ was infected and there were complications; that was the case that surprised me and was how I came to know about the issue. (man not identifying as 'gay' who reported having sex with men)

[It is] a virus that has no cure, it is an illness… that has no remedy, treatment, right? I think that it appears through outbreaks on the hands, like blisters. (Gay sex worker)

I have a cousin that is with papilloma... it is like little bumps that grow… she does not know if it is cancer or papilloma, but they ended up operating on her due to the outbreak… they say it has no cure. (Focus group with gay sex workers)

In contrast, GW were familiar to most participants. Some had seen GW at least once on their sexual partners or clients, while others heard comments about people who had GW:

I have a close friend who this happened to. I believe that they are like warts? Small, skin fragments that stick out. Something like that. (Focus group with gay men)

However, many confused GW with visible or ulcerative STIs, “pimples”, “scars”, “wounds”, and other health problems affecting the anogenital zone, particularly “hemorrhoids”:

When I penetrated a guy he had them, but they were small… one, two [wart(s)] in his anus. Well, I penetrated him with a condom on, right? I did not know what it was… I figured it was a hemorrhoid. (Transgender sex worker)

They are like little water bubbles, I believe they appear in the area of one’s genitals. (Focus group with gay sex workers)

In general, information on HPV and GW came from informal sources (e.g. “rumors”). Four participants who reported they had GW had a better knowledge of HPV and recognized that their GW were sexually acquired, while among subjects without GW only a few recognized the sexual means of transmission. Many participants expressed worry about the possibility of acquiring GW, while others thought that GW were transmitted by “blood” or “lack of hygiene”:

Maybe they don’t have [sex] hygienically… perhaps they are doing it with dirty hands. (Focus group with transgender sex workers)

Two men not identifying as 'gay' who reported having sex with men men considered GW either as a cause or consequence of the immune system’s malfunctioning, and associated the presence of GW with “having AIDS” or “being gay”:

The “queers” get them (Interviewer: Why do you think the “queers” get them?) Sometimes their defenses are weak and they get infected. (Man not identifying as 'gay' who reported having sex with men)

Although some mentioned that GW might produce wounds and bleed, only two people explicitly linked this with the possibility of acquiring HIV:

In the long run it can be dangerous [having GW], because… if the warts were to cut open or get caught on a pubic hair… it can get cut open and it can produce more illnesses, since they are infectious. Both of them are linked to one another [HPV and HIV], because warts can tear. (Gay sex worker)

### Genital wart-related attitudes and experiences

Among the four interviewees who had GW, fear and uncertainty were the predominant feelings associated with discovering GW on their bodies. Due to GW, these subjects experienced stress and distress, embarrassing situations in their sexual lives, as well as physical discomfort (pain, bleeding, discomfort during bowel movements):

[I] felt uncomfortable when I defecated; it hurt when I had sex (…). I felt it was something ugly, for me, I don’t like them, right? And it is something uncomfortable. (Transgender sex worker)

[The GW] grow, stick out, and end up bleeding by rubbing against underwear fabric. They hurt a lot. They appeared on my penis… I thought it was something from my prostate, something internal that was bleeding, and I didn’t pay attention to the pain, but the crude reality… I looked at them up close… they were genital warts. (Gay sex worker)

Participants with GW avoided disclosing to their sexual partners that they had GW in order to prevent rejection, and feared transmitting their GW to others:

(I: Do you normally tell your sex partners about your infection with palilloma?) No. (I: Why not?). Because…I don’t know. I just don’t tell them. (Gay sex worker)

[When the GW appeared] … I got very scared and I did not know what to do. I stopped having sex because I was embarrassed and I was afraid of infecting others. (Gay man)

One participant stopped having sex when he discovered his GW, and other said he changed his sex role (from passive to active) in order to conceal his anal warts:

I liked it when men play with that area [anus] and now they cannot. One [man] made me feel bad, he asked: “What happened to you there?” He was going to rim me, but he lost the desire to do so and I felt ashamed… and I never saw him again. [Later] I even became [the] active [partner]… because I didn’t want them touching my backside. (Gay man)

Although most participants in discussion groups initially said they had never seen GW, some recognized them after seeing the images of GW presented in the study. Upon viewing the GW images, many participants visibly reacted (e.g. expressing repulsion):

Right now that I see them [pictures of GW] on the screen, the truth is that I feel somewhat bad, um… a bit uncomfortable. The truth is, looking at the picture, I feel a bit tense. (Gay man)

The pictures that were there were nasty [laughs]! Ick! Disgusting! Those [GW] look really nasty in those photos, I’ve never had that. (Man not identifying as 'gay' who reported having sex with men)

The transgendered participants were less uncomfortable and notably most familiar with GW; they even referred to them using nicknames such as “grapes”, “earrings”, or “gizzards”:

As a transgendered, usually the top guys pick me up… but when I was [sexually] versatile I saw the real “grape harvest” that they had there, the real “grapes”. (Transgender sex worker)

Those [GW] are the “little earrings” they have. (Focus group with transgender sex workers)

Among most transgendered people GW were seen as bothersome and a source of mockery, but for other groups GW were not a theme of conversation among peers, couples, or clients.

Some participants reported that they had seen GW in their sexual partners, and mentioned having experienced astonishment and repulsion, embarrassing situations, distrust and fear of becoming infected. In these cases, sex was frequently interrupted:

I have seen it [GW] on some occasional partners… I’ve seen that they are like little warts in the anus; and I said: “I’m not getting close to that.” (Focus group with gay men)

I was groping around and there was a wart and… I felt something ugly like a think mole, a meaty, raised mole… I lost all interest… it grossed me out. (Focus group with gay sex workers)

A guy told me that he saw some little bumps in a queers ass and didn’t want to penetrate him and only let him give oral sex. He told me that he was disgusted but didn’t do anything with the other guy’s ass. (Man not identifying as 'gay' who reported having sex with men)

People with GW tried to conceal them (e.g. by having sex in darkness) due to shame or denied having GW or justified their presence by saying they were “hemorrhoids”, “moles”, “scars” or “burns”:

[A client] turned the lights off on me. I suspected that something wasn’t right, so I turned on the light and he… had removed the condom… I carefully checked him out and I saw a fleshy white growth… I didn’t know if it was papilloma… I asked him, “What do you have there?” “Nothing,” he said, “it is a burn” “That’s not a burn,” I said, “A burn doesn’t get like that.” immediately kicked him out. (Transgender sex worker)

### Management of genital warts

Self-management of GW as an alternative to medical intervention was reported. Some transgendered participants discussed self-management procedures aimed to excise GW by using “razor blades”, “scissors”, “pubic hairs” (to make “noose” around the GW and cut them) and “hands”:

[One GW] moved like a little worm. I think [a friend] cut it off using his hand… (Another FG participant) Same here, I cut it off but it bled a lot so I covered it with cotton. (Focus group with transgender sex workers)

These solutions were recognized as only temporary and implicating a risk of infection. One transgendered participant mentioned that she sometimes helped a friend to remove GW with these methods:

I had a younger [female] friend… I cut it off with small scissors… [one GW] was stuck in her anus. (Focus group with transgender sex workers)

Two interviewees affected by GW looked for medical support soon after noting they had GW. Others looked for medical help after having attempted self-management/medication, and, later, after their GW grew, spread or became a serious problem:

Some little warts appeared on my backside and I was a little bit embarrassed to have them checked out, and they grew a lot, and then when they bothered me, I told a friend and [he or she] said to go to a hospital. (Gay man)

In all cases, access to medical treatment was mediated by friends and health promoters:

I only used a cream from the pharmacy initially, and then nothing happened. Then from there one day I talked to a friend who worked as a health promoter. She was the one who took me to [the health center]. (Transgender sex worker)

One participant mentioned that he found help only after three unsuccessful attempts at health care establishments where the medical personnel didn’t know how to manage GW or didn’t have the instruments for cauterization:

There is no medical specialist for that… two physicians told me ‘men do not get papilloma’ [In another health center the doctor] gave me a prescription for treatment, a cream, but I couldn’t find it and was very expensive… it did not exist in the pharmacies. [Afterwards,] I talked to a friend that is also a doctor, and he said ‘those are genital warts’ and he wanted to cauterize them but did not have the equipment. (Gay sex worker)

Some focus group participants, mainly transgenders, discussed cases of friends who had GW but didn’t seek treatment, which they attributed to “shame”:

I asked [my friend] ‘Hey, what do you have there?’ And she didn’t even know because [she is] young, 19 years old. I told to her that she had to be checked out, but I don’t know, even now she doesn’t pay attention to me, she hasn’t gone to get checked out… I think is because of embarrassment. (Transgender sex worker)

## Discussion

This study is the first in-depth, qualitative assessment on HPV-related GW in South American MSM and transgendered women. We found that while participants were unfamiliar with HPV, experiences with GW were relatively common. Most participants did not differentiate between GW and other health problems affecting the anogenital zone, and two participants recognized that lesions associated with GW could represent an increased HIV risk. GW profoundly affected the emotional, social and sexual lives of those who had them, including the ability to find competent medical care. Our data show how misinformation and GW-related stigma synergize to produce social rejection toward those who have GW, and limit their search for treatment. A particular concern arises among self-treatment of GW which could result in anogential wounds or infection, which in turn could exacerbate the risk for HIV infection.

Our study results support findings from previous work showing the limited HPV knowledge among men and women from several countries [25]–[27], including MSM from the US, Denmark, Australia and [16], [28]–[31]. Overall, our data coincide with several studies indicating that most men are unaware that GW are caused by HPV infection [13], [30], [32]–[33]. Although some reports have documented the negative impact of GW on individuals’ quality of life, sexuality and psychological status [12], [16]–[19], [34]–[38], few qualitative studies have employed in-depth approaches to understand the experiences of men with GW [12], [15], [18]–[19], [34], [36], and none have obtained the particular views of South American transgendered women, sex workers or people without GW on this health problem, although a high HPV prevalence has been found in these groups in Argentina, Brazil and Peru [4]–[5], [39].

We have identified new information that is useful for health professionals and researchers devoted to GW care and prevention in Peru, namely, the GW self-management practices and the limitations for accessing GW-specific health care. In this study, medical treatment was important to all participants but navigating the medical system was somewhat difficult, while a gap in the knowledge on HPV/GW management was also apparent among health professionals. These issues may be common in other countries where the health systems face resource constraints. The important role of peers facilitating access to treatment in Peru may be applicable to other environments as well.

Individuals who reported not having GW had strong concerns and fear of acquiring them, as well as stigmatizing attitudes toward people with GW. That being said, it’s not surprising that people affected by GW try to strategically conceal them by saying they have “hemorrhoids”, “moles”, “scars” or “burns” since attributing an anogenital lesion without an STI origin avoids moral judgment on sexual behavior.

This study had limitations. Since GW is a theme surrounded by shame and stigma, some participants may have concealed that they had GW or felt uncomfortable disclosing their own experiences in front of peers or study facilitators. In the focus groups we expected to receive socially accepted answers, and data given by participants in the individual interviews were likely subject to social desirability bias, as well. In addition, our convenience sample included only a small number of subjects and did not reach all forms of sexual diversity present in Peru within the MSM and TG categories. Thus, other groups (e.g. “closeted” gay, or clients of male sex workers) may likely have different views of GW. In fact, we have shown that attitudes and experiences differed between transgendered persons and other groups and are likely to be different in the MSM sub-groups as well.

These findings highlight the importance of GW-related stigma, the involvement of peers as facilitators of access to prevention and treatment, and the link between HPV/GW and HIV risk among Peruvian MSM. Future STI/HIV programs focused on high risk groups including MSM/TG should be aware of these issues. Likewise, broader educational efforts will be also necessary in order to increase the public awareness of HPV infection and its sequelae.

Finally, our study results call attention to the need for additional research on HPV among MSM/TG from Peru and other South American countries. Data on HPV prevalence, behavioral risk factors for HPV transmission, cancer screening, medical skills for HPV/GW prevention and management, and relationships between HPV and other STI are needed. A better understanding of the HPV burden in MSM/TG will help identify if routine cancer screening, HPV testing, and HPV vaccination campaigns should be a priority for these populations.
